# “Influence of macroretention in polymer-infiltrated ceramic network- and zirconia crowns and different roughness values of titanium bases on adhesive bond strength of hybrid abutment crowns: an in vitro study”

**DOI:** 10.1186/s12903-026-09606-7

**Published:** 2026-08-15

**Authors:** Leonie Grander, Annika Kraus, Felicitas Hölken, Timpe Heidebrecht, Lisa Steiner, Samir Abou-Ayash, Stefan Wentaschek

**Affiliations:** 1 Department of Prosthodontics and Biomaterials, University Medical Center Mainz, Augustusplatz 2, 55131 Mainz, Germany; 2 Department of Reconstructive Dentistry and Gerodontology, School of Dental Medicine, University of Bern, Freiburgstrasse 7, Bern, 3007 Switzerland

**Keywords:** Hybrid abutment crown, Tensile strength, Bond strength, Titanium base, Polymer-infiltrated ceramic networks

## Abstract

**Background:**

Two-piece hybrid abutment crowns (HACs) are CAD/CAM-fabricated implant restorations adhesively bonded to prefabricated titanium bases (Ti-bases). Since adhesive bond represents a potential weakness, methods to enhance long-term durability are being investigated. The aim of this in vitro study was to evaluate the influence of additional macroretention, specifically a circumferential groove, incorporated into polymer-infiltrated ceramic network (PICN) and zirconia crowns, and two Ti-base sandblasting protocols on adhesive bond strength of HACs.

**Methods:**

Ninety Ti-bases, 45 zirconia and 45 PICN crowns were divided into six groups (*N* = 15). Prior to conventional cementation (Panavia V5, Kuraray) of zirconia control group (Z1), Ti-bases and internal crown surfaces were sandblasted (Al₂O₃; 50 μm; 1.0 bar) and treated with MDP primer. In PICN control group (H1), Ti-bases were sandblasted identically and pretreated with Adiva M-Prime. Internal surfaces of PICN crowns were etched (5% hydrofluoric acid) and primed with Adiva C-Prime. In Z2 and H2, a circular groove in the internal crown surface was prepared as a macroretention in addition to conventional pretreatment as performed in Z1 and H1. Z3 and H3 were also conditioned conventionally, as in Z1 and H1, but Ti-bases were sandblasted with 250 μm at 2.0 bar. After sandblasting, surface roughness values (RV) of Ti-bases were measured. Following thermocycling, tensile shear test was performed, and failure mode was analysed. Mean values were compared using t-test.

**Results:**

RV differed significantly between differently blasted Ti-bases (*p* < 0.001). Both crown materials achieved significantly higher mean pull-off forces (Z1: 1085 *N* ± 79; H1 = 664 *N* ± 68) in control groups than in groups with macroretention (Z2: 1017 *N* ± 65; H2 = 438 *N* ± 48) (Z: *p* = 0.024, H: *p* < 0.001), and significantly lower pull-off forces than in groups with rougher Ti-bases (Z3: 1298 *N* ± 98, H3: 744 *N* ± 64) (Z: *p* < 0.001, H: *p* = 0.0023).

**Conclusions:**

Increasing pressure and grit size during corundum blasting of Ti-bases can achieve higher pull-off force for zirconia and PICN crowns. Additional macroretention, at least in the form used here, offers no advantage regarding bond strength.

## Background

Hybrid abutment crowns (HACs) are CAD/CAM-fabricated implant restorations adhesively bonded to prefabricated titanium bases (Ti-bases). Advantages of CAD/CAM manufacturing include access to prefabricated materials processed under controlled industrial conditions, improved precision and planning, enhanced reproducibility, and more efficient data storage. In digital implant dentistry, HACs are employed for both single tooth replacement and more extensive rehabilitations. While the Ti-base is mechanically connected to the implant by a screw, the connection between Ti-base and abutment crown occurs exclusively through adhesive bonding. A stable bond between Ti-base and ceramic crown is essential for long-time restoration success. To ensure bond stability, adhesive selection and component geometry must be optimally coordinated. Moreover, the bond must withstand the oral cavity’s long-term thermal, chemical, and mechanical stresses [1–4]. To optimize bond strength, both crown and Ti-base are commonly pretreated according to standardized protocols differing between materials, but all aim to clean, roughen, and activate the surface via chemical, mechanical, or mechanochemical processes [5–7]. Gold-standard pretreatment for zirconia crowns is airborne-particle abrasion/ sandblasting (SB) with alumina (Al_2_O_3_; 50 μm grit size) at 10 mm distance and 1 bar pressure, followed by application of amphiphilic 10-Methacryloyloxydecyldihydrogen-phosphate (10-MDP) primer [8–11]. For ceramic compositions such as Vita Enamic (polymer-infiltrated ceramic network (PICN); ceramic network 86 vol%, polymer 14 vol%), investigated in the present study, standard pretreatment involves etching with 5% hydrofluoric acid (HF) for 60 s, followed by silane application [12, 13]. The bonding surface of Ti-bases is routinely SB with alumina (50 μm grit size) at 10 mm distance, followed by the application of a bonding agent [3]. Recommendations for titanium conditioning are inconsistent. Reported alumina grit sizes range from 50 to 250 μm [5] and blasting pressures from 1.0 to 4.0 bar [14, 15]. The manufacturers of the Ti-bases often recommend a grit size of 50 μm and a maximum blasting pressure of 2 bar. Despite widespread adoption of this largely standardized conditioning process, adhesive bond failure at the Ti-base-crown interface still occurs in clinical practice. Adhesive bond failure can be classified into distinct failure modes. In cohesive failures, the fracture occurs within the adhesive material itself, leaving adhesive on both opposing surfaces. In adhesive failure, the bond fails on one of the two surfaces, thus indicating which surface had a weaker bond. In previous studies performing bond strength tests with HACs, exclusively adhesive failures occurred [16, 17]. These manifested either as complete adhesive detachment from the ceramic, with adhesive fully remaining on the Ti-base, or as mixed failures in which residual adhesive also persisted partly on the ceramic substrate. Mean pull-off force always was highest when at least some of the adhesive material remained on the ceramic [16, 17]. These results indicate that the adhesive interfaces to zirconia and PICN constitute the weakest connections, and that enhancing retention at the crown’s internal surface is essential for increasing overall bond strength. Given limitations on blasting pressure and grit, particularly for ceramics [18, 19], this study examined whether adding a macroretention (MR) in PICN and zirconia crowns, or using a larger-than-recommended alumina grit size on the titanium surface, could improve adhesive bonding. The null hypotheses are that pretreatment protocol has no influence on the pull-off force (F_max_) between crown and Ti-base, that fracture behaviour is not related to F_max_, and that alumina grit size has no influence on the roughness parameters of the Ti-base.

## Methods

### Preparation of crown specimens

**Fig. 1:**
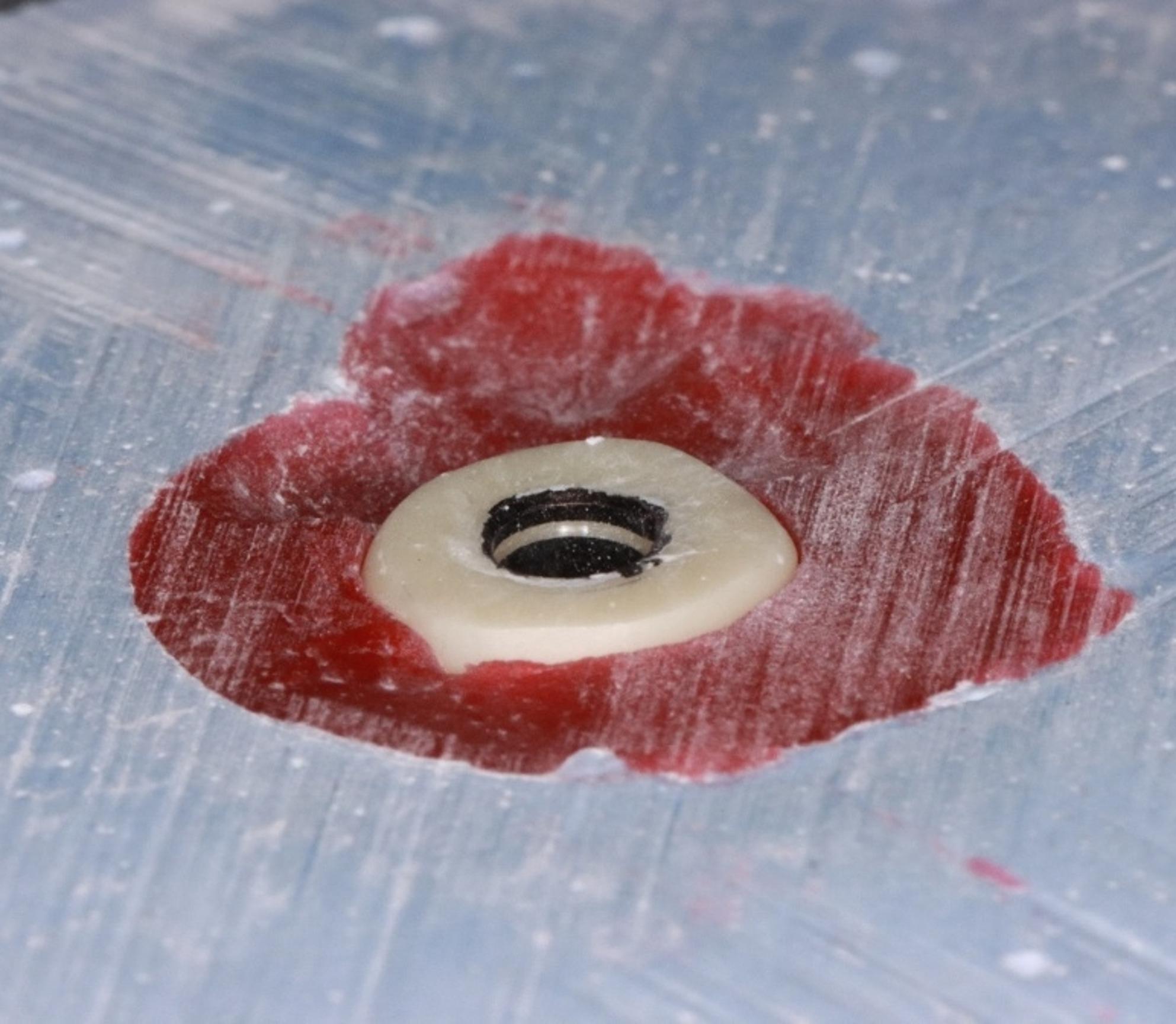
Zirconia crown base with MR (internal circumferential groove)

**Fig. 2:**
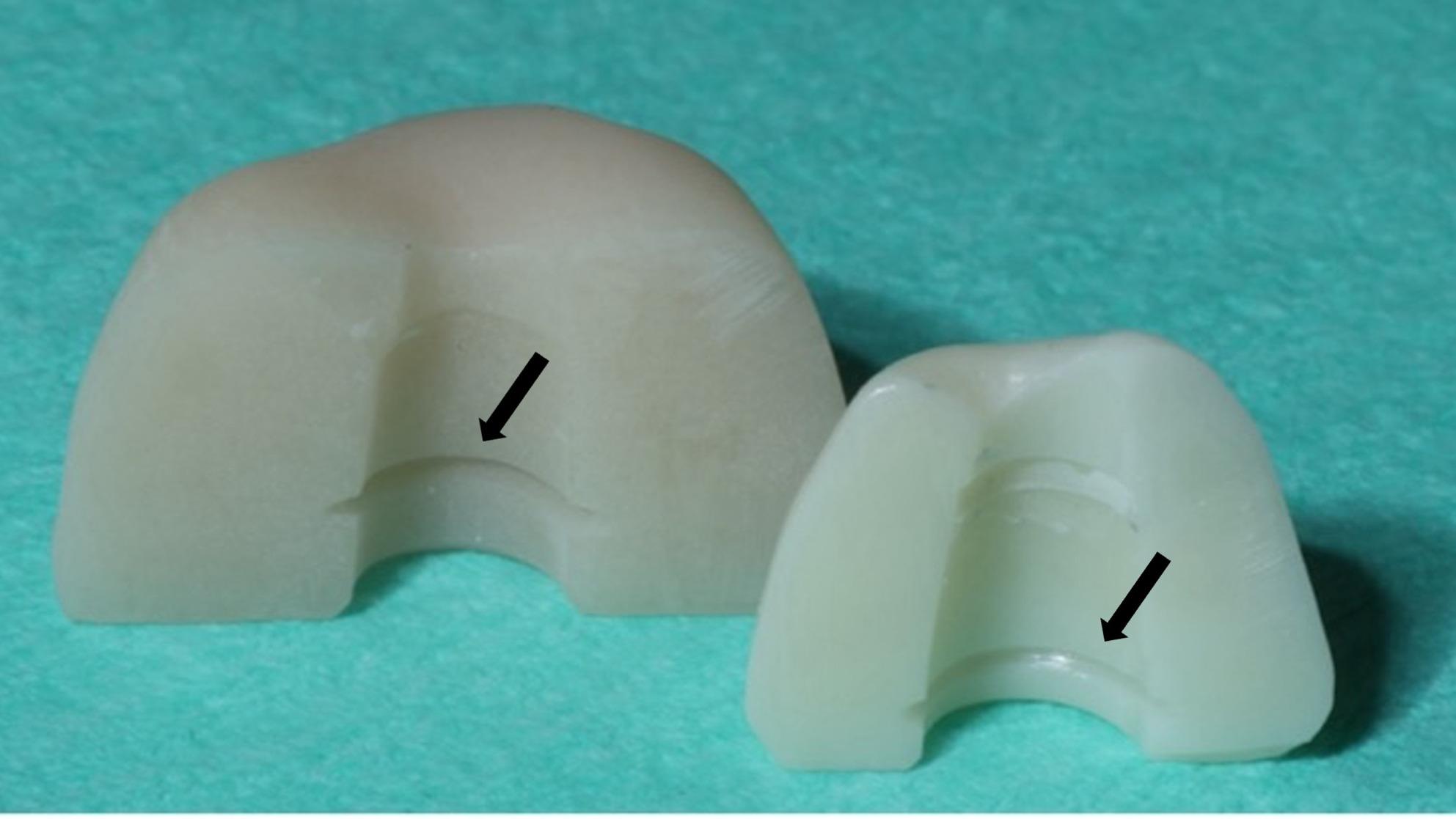
Zirconia (right) and PICN (left) crown sectioned through center with MR

Ninety Ti-bases (Ti-Base NB RS 4.3 L, Sirona Dental Systems GmbH, Bensheim, Germany), 45 Vita PICN crowns (VITA ENAMIC^®^ for CEREC^®^/inLab, VITA Zahnfabrik, Bad Säckingen, Germany) and 45 zirconia crowns (inCoris ZI meso F2 L, Sirona Dental Systems GmbH, Bensheim, Germany) were divided into 6 groups containing 15 two-piece HACs each. Both HACs were designed using CEREC SW^®^ 5.0 software (Sirona Dental Systems GmbH, Bensheim, Germany). Blocks of both materials featured a manufacturer-prefabricated internal connection for the Ti-base to ensure uniform fit and cement gap size. Moreover, no measurements of the cement gap size were performed, as it was assumed to be uniform based on the manufacturer’s preconfigured design, to the extent permitted by manufacturing tolerances. Crowns were designed to have a minimum thickness of 3 mm around the screw hole. The design was positioned in the milling block with the lower part extending beyond the block to provide a flat bearing surface for the pull-off device. Crowns were fabricated using CEREC MC XL milling unit. The zirconia crowns milled from pre-sintered blocks were sintered according to the manufacturer’s instructions. Crown design and testing method replicated those employed in prior studies [16, 17]. Before further processing, 15 PICN (H2) and 15 zirconia (Z2) crowns were provided with a MR in the form of a circumferential groove in the bonding surface within the prefabricated Ti-base recess (Fig. 1) by securing the crowns in a parallel milling machine and grinding the internal surface in an annular pattern with a lens-shaped diamond bur (REF 825.104.050, Komet) under continuous water cooling (Fig. 2). The groove was located 2 mm from the flattened underside for zirconia and 3 mm for PICN crowns.

After MR was applied, all crowns were conditioned and cemented using the protocol recommended by the manufacturer. Adhesive surfaces of PICN crowns in H1, H2, and H3 were etched with HF (VITA Ceramics Etch 3 ml syringe, VITA Zahnfabrik, Bad Säckingen, Germany) for 60 s. HF was removed completely with running water and a steam jet device (Triton SLA, Bremer Goldschlägerei Wilh. Herbst GmbH & Co. KG, Bremen, Germany). PICN crowns were dried with oil-free, clean air and treated with silane bonding agent (VITA ADIVA C-Prime, Harvard Dental International GmbH, Hoppegarten, Germany) for 10 s directly before bonding with Ti-bases. Inner adhesive surfaces of zirconia crowns were pre-coloured using a black marker to ensure uniform processing before MR was milled in Z2 (Fig. 1) and before SB of the inner surfaces of Z1, Z2 and Z3. Zirconia bonding surfaces were SB using a dental sandblaster (P-G 400, Harnisch+Rieth GmbH & Co. KG, Winterbach, Germany) with 50 μm alumina (Al_2_O_3_) (Plurakorund, Pluradent AG & Co. KG, Offenbach, Germany) at 1.0 bar pressure and 10 mm distance for 10 s (Z1, Z2, Z3). After SB, zirconia crowns were cleaned with 96% ethanol in an ultrasonic cleaner for 5 min. Directly preceding bonding with Ti-bases, 10-MDP primer (Clearfil Ceramic Primer Plus, Kuraray Medical Inc., Okayama, Japan) was evenly applied to the bonding surface and carefully blow-dried after 10 s (Groups Z1, Z2, Z3).

### Preparation of Ti-bases

SB of titanium surfaces was performed with Al_2_O_3_ (Plurakorund, Pluradent AG & Co. KG, Offenbach, Germany) at approximately 10 mm distance and 90° angle. For H1, H2, Z1 and Z2, a grit size of 50 μm at 1.0 bar was used, and a grit size of 250 μm at a pressure of 2.0 bar for H3 and Z3. After SB, surface roughness of all Ti-bases was determined. As only sandblasted Ti-bases were used, roughness measurement was conducted only after SB using a surface roughness measuring device (Perthometer PRK, Mahr). Three individual measurements were taken for each abutment over a measuring distance of 1 mm each. The measurement was performed axially at a feed rate of 1 mm/s. The average value for each parameter was calculated separately from the three individual measurements of each abutment. The roughness parameters determined in µm were Ra (arithmetic mean roughness), Rq (quadratic mean roughness) and Rz (average roughness depth). After roughness measurement and immediately before final bonding, Ti-bases were conditioned with system-compatible primers. Therefore, a thin layer of bonding agent was applied with an applicator brush and carefully blow-dried after 10 s. In H1, H2 and H3, VITA ADIVA M-Prime (VITA ADIVA M-Prime, Metal/Alloy primer, Harvard Dental International GmbH, Hoppegarten, Germany) was used as a primer, while Z1, Z2 and Z3 were treated with Clearfil Ceramic Primer Plus (Kuraray Medical Inc., Okayama, Japan). Table 1 summarizes the pretreatment protocols of all groups.

**Table 1:** Overview of performed pretreatment protocol for each group

	Pretreatment of crown (*N* = 90)	Pretreatment of Ti-base (*N* = 90)	Adhesive system (*N* = 90)
Group H1 (*N* = 15)	PICN + HF + C-Prime	50 μm (1 bar) + M-Prime	Adiva IA
Group H2 (*N* = 15)	PICN + MR + HF + C-Prime	50 μm (1 bar) + M-Prime	Adiva IA
Group H3 (*N* = 15)	PICN + HF + C-Prime	250 μm (2 bar) + M-Prime	Adiva IA
Group Z1 (*N* = 15)	Zirconia + SB 50 μm (1 bar) + Clearfil	50 μm (1 bar) + Clearfil	Panavia V5
Group Z2 (*N* = 15)	Zirconia + MR + SB 50 μm (1 bar) + Clearfil	50 μm (1 bar) + Clearfil	Panavia V5
Group Z3 (*N* = 15)	Zirconia + SB 50 μm (1 bar) + Clearfil	250 μm (2 bar) + Clearfil	Panavia V5

### Adhesive bonding

Depending on crown material, pre-treated components were bonded with VITA ADIVA IA-CEM (Harvard Dental International GmbH, Hoppegarten, Germany) (Group H1, H2, H3) or Panavia V5 (Kuraray Medical Inc., Okayama, Japan) (Group Z1, Z2, Z3). Ti-bases remained screwed onto the laboratory implant (Nobel Parallel Conical Connection RP 4.3 × 11.5 mm, Nobel Biocare Services AG, Zürich, Switzerland) and secured in a plastic clamp (Mediplast AB, Malmö, Sweden). The screw channel was sealed with cotton. Bonding agents were mixed using auto mix cannulas (3 M Deutschland GmbH, Neuss, Germany), with the first 5 mm of dispensed material being discarded to ensure a homogeneous mixture. The luting composite was subsequently applied as a uniform layer to the external surface of the Ti-base and the internal surface of the ceramic crown using a fine application brush. The components were seated precisely, and excess material inside the screw channel was removed with microbrushes. To ensure consistent bonding pressure during polymerization, the bonded abutments were secured in a hybrid abutment bonding aid (HPdent GmbH, Gottmadingen, Germany). After partial light curing along the bond line for 5 s from all directions with a light curing lamp (1200 mW/cm^2^, Elipar S10, 3 M Deutschland GmbH, Neuss, Germany), excess material was removed with a LeCron spatula (Henry Schein Dental GmbH, Langen, Germany). The bond line was subsequently covered with glycerin gel (Liquid Strip, Ivoclar Vivadent AG, Schaan, Liechtenstein), followed by full light curing for 40 s from all sides. After a 10-minute chemical curing phase, the bonding aid and glycerin gel were completely removed. Final curing was completed at room temperature for at least 24 h, after which the adhesive margin was polished. All procedures with both materials were performed using gloves to prevent contamination. To simulate aging, the bonded HAC´s were subjected to thermocycling (Thermocykler Willytec, SD Mechatronik GmbH, Feldkirchen-Westerham, Germany) between distilled water baths at 5 °C and 55 °C for 5000 cycles. Each cycle included a 30-second dwell in each bath and 10 s of dripping- and-transfer time between baths. This thermocycling protocol intends to simulate an approximate clinical service period from six months to one year and has also been employed in comparable studies [20, 21]. Specimens were stored in distilled water at room temperature (23 °C) before and after thermocycling until further processing. All specimens were prepared in the prosthetic laboratory of the university medical centre, while bond strength testing (and surface roughness measurements) was performed in a blinded manner by a different operator in a separate materials science laboratory.

### Bond strength testing

Bond strength tests were performed using a universal testing machine (Zwick Z005, Zwick/Roell). F_max_ in Newton (N) required for complete crown removal or failure of the composite adhesive bond was recorded. To prevent false triggering due to short-term load fluctuations, the failure cutoff was set to 50 N. For axial force application, crowns were screw-tightened to the laboratory implant with 35 Newtoncentimeter (Ncm), which was then fixed in a wedge-shaped specimen holder mounted in the lower part of the machine. The flat surface of the crown rested on a polished 1-mm-thick steel disc with a central opening for the implant. The specimen holder’s fully articulated mounting ensured a centered, exclusively axial load along the implant-HAC-axis. Load was applied at a feed rate of 1 mm/min until complete failure of the adhesive bond. After completion of the bond strength tests, specimen fracture surfaces were examined using a light microscope (VHX-1000, Keyence Deutschland GmbH, Neu-Isenburg, Germany) at 30x magnification. Failure mode was assessed visually and qualitatively classified into the following categories:

- Adhesive failure on the titanium surface: Adhesive remaining entirely on the ceramic.
- Adhesive failure on the ceramic surface: Adhesive remaining entirely on the Ti-base.
- Combined adhesive failure: Adhesive remaining on both the titanium and ceramic surfaces.
- Cohesive failure: Failure within the adhesive layer with material remaining on both corresponding sides of the bonded components.

### Statistical analysis

Statistical analysis was performed using SPSS Statistics software (SPSS Statistics Version 27, IBM Corp., Armonk, New York, USA). Before conducting inferential analyses, underlying statistical assumptions were verified. The normal distribution of the data within groups was tested using Shapiro-Wilk test, while homogeneity of variance between groups was examined through Levene’s test. If assumptions were met, independent-samples t-tests were used; in cases of unequal variances, Welch’s t-test was applied. For groups deviating from normality, Mann-Whitney U test was performed. In addition to p-values, 95% confidence intervals were calculated. As the study is an exploratory in vitro study, no a priori sample size calculation was performed. The effect sizes were determined post hoc via sensitivity analysis (two‑sample independent t‑tests, two‑tailed, α = 0.05, 80% power) in G*Power 3.1 [22]. Cohen´s d was calculated as d = 1.0598. The significance level was set at α = 0.05 for all analyses.

## Results

With Cohen´s d = 1.0598, minimal detectable differences of pull-off values between 62.4 N and 94.4 N were calculated using pooled standard deviations. In groups Z2 (*p* = 0.04) and H2 (*p* = 0.047), pull-off values showed a significant deviation from normality. No significant deviations from normality were observed for pull-off values in the remaining groups or for roughness values in all groups (*p* > 0.05). Homogeneity of variance was met for pull-off values and Rz values (*p* > 0.05). However, significant differences in variance between groups were found for the roughness parameters Ra (*p* = 0.02) and Rq (*p* = 0.02).

### Roughness values

Baseline roughness parameter values measured after conducting the two different SB protocols are presented in Table 2. Within each group, Ti-bases showed comparable roughness values (Table 2). The two investigated different blasting procedures significantly affected all evaluated roughness parameters of the Ti-bases in comparison between groups (*p* < 0.001 in each case, Ra: d = 4.04; Rq: d = 4.16; Rz: *r* = 0.81).

**Table 2:** Mean roughness parameters with minimum, maximum and standard deviation of titanium abutments after SB with 50 μm Al_2_O_3_ and 1.0 bar pressure vs. with 250 μm and 2.0 bar pressure

Roughness parameters	50 μm, 1 bar (*N* = 60)	250 μm, 2 bar (*N* = 30)
Ra	2.30 (Min. 1.9, Max. 2.8, SD 0.21)	3.38 (Min. 2.8, Max. 4.2, SD 0.36)
Rq	2.94 (Min. 2.4, Max. 3.5, SD 0.27)	4.37 (Min. 3.5, Max. 5.4, SD 0.45)
Rz	16.99 (Min. 14.1, Max. 21.8, SD 1.8)	24.14 (Min. 22.2, Max. 29.3, SD 1.65)

### Bond strength tests

Mean pull-off forces for the six groups are presented in Table 3. The zirconia groups exhibited mean pull-off force (Fmax) values between 1017 *N* ± 65 N and 1298 *N* ± 98 N, whereas the PICN groups showed values between 438 *N* ± 48 N and 744 *N* ± 64 N.

**Table 3:** Mean pull-off values of all groups with minimum, maximum and standard deviation

Group	*N*	Mean [*N*]	Min. [*N*]	Max. [*N*]	SD [*N*]
Z1	15	1085	950	1220	79
Z2	15	1017	950	1147	65
Z3	15	1298	1099	1452	98
H1	15	664	553	778	68
H2	15	438	386	561	48
H3	15	744	641	850	64

**Fig. 3:**
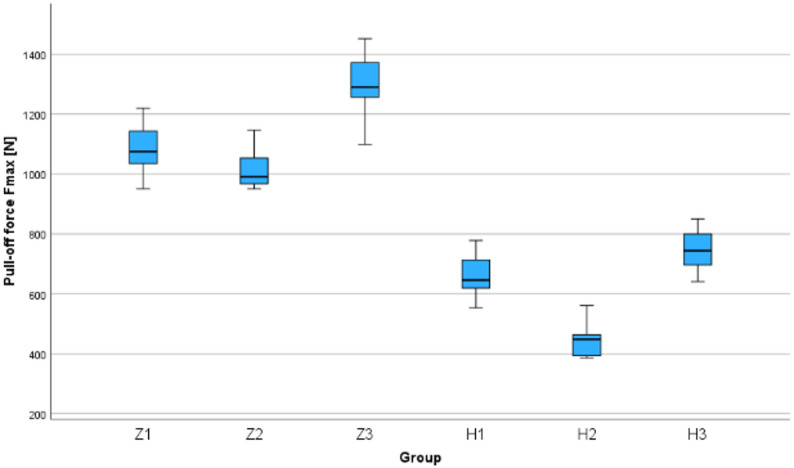
Boxplot representation of pull-off values for zirconia and PICN crowns

For both zirconia and PICN crowns, the corresponding MR groups (Z2 and H2) exhibited significantly lower pull-off values (Z1 vs. Z2 *p* = 0.024, *r* = 0.42; H1 vs. H2 *p* < 0.001, *r* = 0.84) and the groups with rougher Ti-bases (Z3 and H3) achieved significantly higher pull-off values (Z1 vs. Z3 *p* < 0.001, d = 2.40; H1 vs. H3 *p* = 0.0023, d = 1.22) than the corresponding control groups (Z1 and H1) (Fig. 3).

### Failure mode analysis

**Fig. 4:**
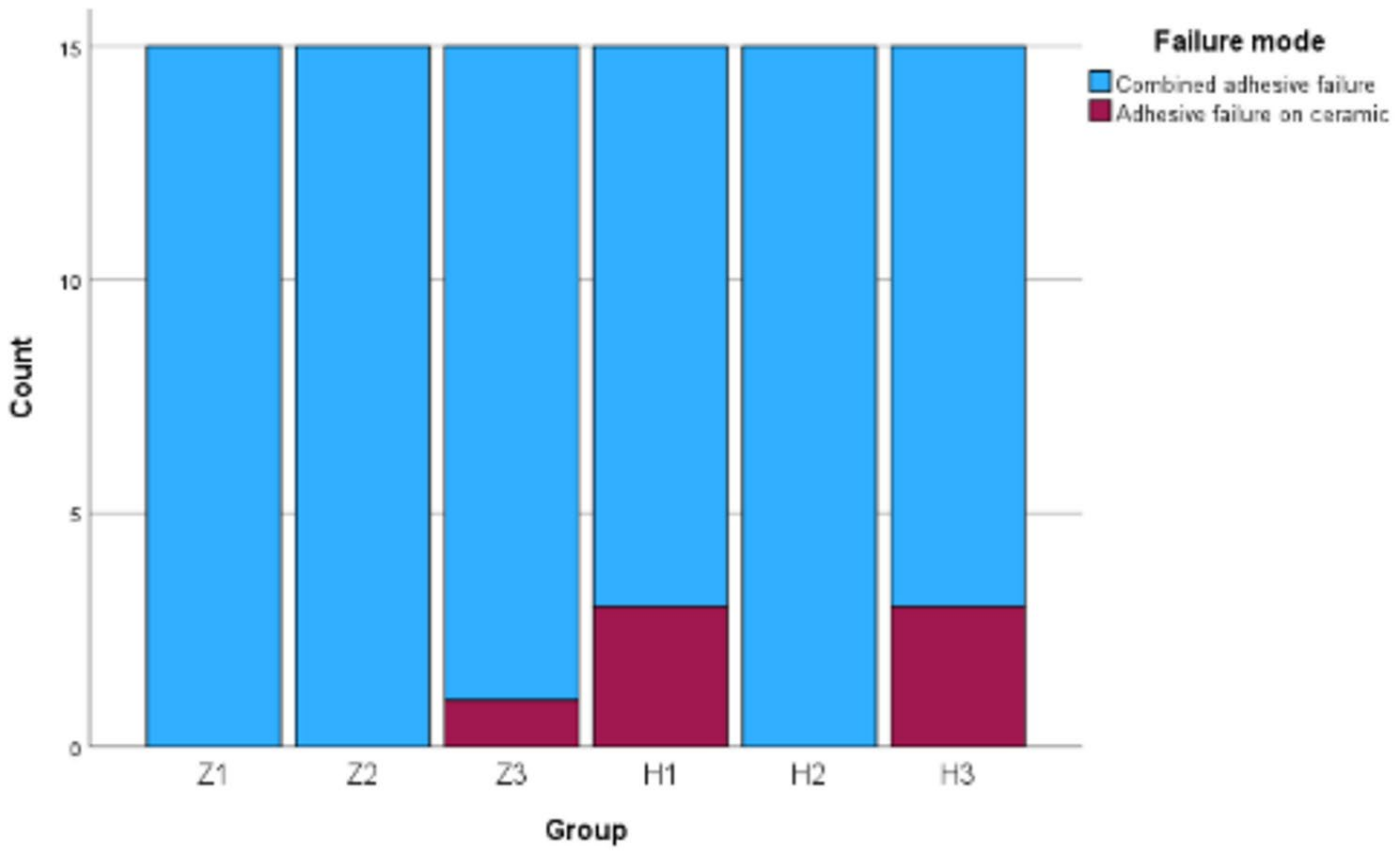
Bar chart to illustrate the distribution of failure mode

Of the 90 specimens, 83 exhibited combined adhesive failures with adhesive remaining on both the titanium and ceramic surfaces, whereas seven specimens demonstrated exclusively adhesive failures at the ceramic interface, with adhesive remaining entirely on the Ti-base (Fig. 4). Exclusively adhesive failure of the titanium and cohesive failure did not occur. No correlation with the pull-off force could be demonstrated (*p* = 0.306, *r* = 0.11).

**Fig. 5:**
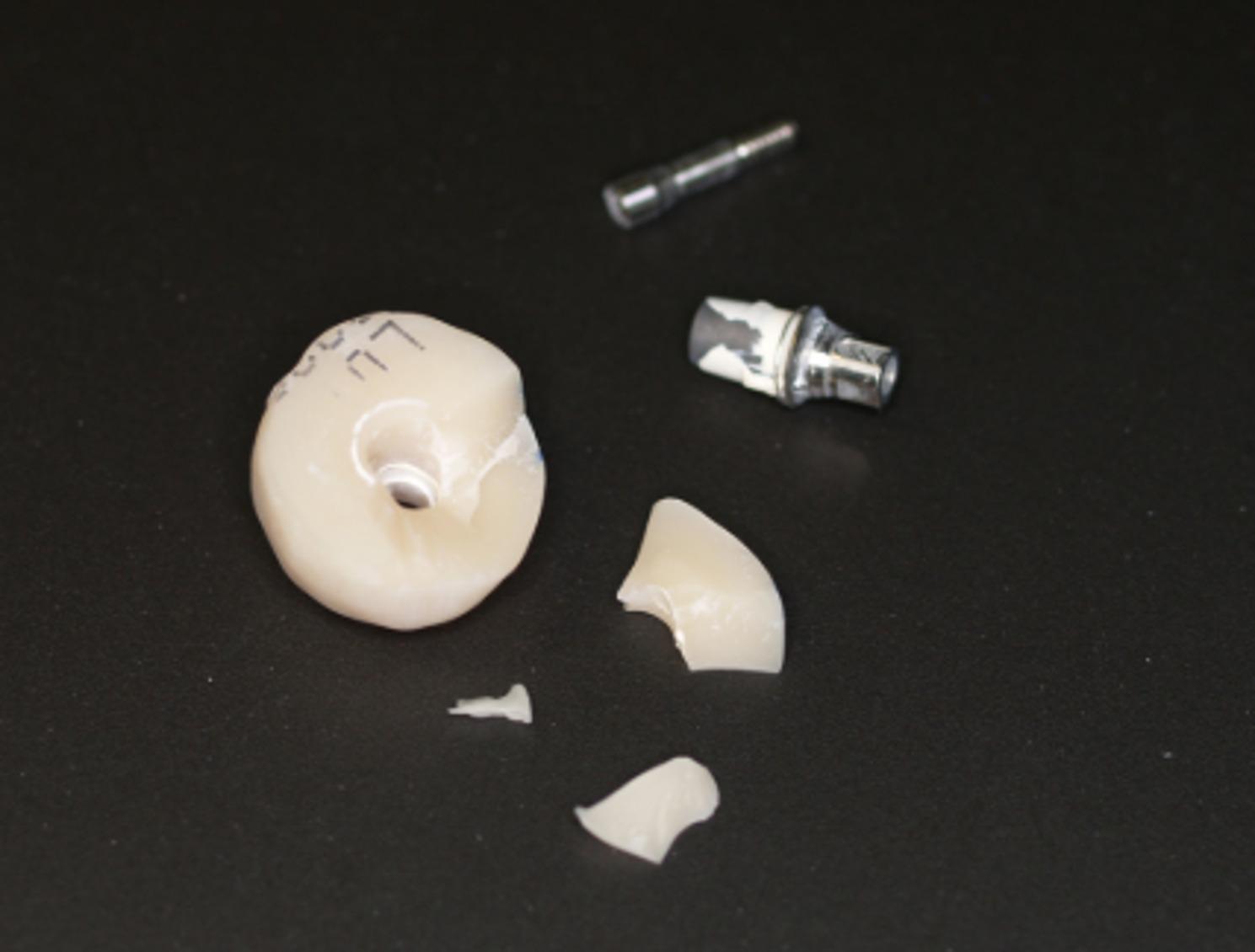
PICN crown after pull-off test with combined adhesive failure and fracture in the area of the bearing surface

Despite the non-anatomical crown design, in which a large, flat bearing surface was created for the pull-off tests, fragments broke off in the bearing area before the PICN crowns fully detached from the Ti-bases (Fig. 5), occurring in four crowns in group H1, five in group H2, and five in group H3.

## Discussion

The aim of this in vitro study was to investigate the influence of additional MR and modified SB parameters on bond strength of zirconia and PICN crowns bonded to prefabricated Ti-bases. Two modifications of the bonding system were evaluated: incorporation of MR in form of a circumferential groove on the internal ceramic surface and increased particle size and pressure during Ti-base SB. It was also investigated whether fracture behavior was related to pull-off force. For both crown materials, incorporation of internal MR in addition to the standard protocol did not lead to an improvement, but rather to a reduction of mean pull-off force. Groups with rougher Ti-bases after SB achieved higher pull-off values than corresponding groups, where SB was performed according to standard protocol. A correlation between fracture pattern and pull-off values could not be proven. Accordingly, the null hypotheses asserting that pretreatment has no influence on bond strength between crown and Ti-base and that particle size and blasting pressure have no effect on Ti-base roughness parameters were rejected. The null hypothesis positing no association between fracture behavior and pull-off force was retained.

The calculated effect sizes were large in both material groups, indicating a strong and clinically relevant influence of this surface modification. Analysis of failure modes predominantly revealed mixed adhesive failures. Pure cohesive failures within the luting composite were not observed, indicating that the intrinsic strength of the composite exceeded the adhesive strength at the interfaces. The reduced pull-off force observed following incorporation of a MR in the ceramic crowns can be attributed to the modification of the adhesive interface geometry induced by the groove. The resulting increase in adhesive layer thickness at the exact location of the MR that could be assumed after adding an additional groove, even if it was not explicitly measured, may have led to enhanced polymerization shrinkage, thereby inducing microcracks or gap formation, adversely affecting bond strength. Given that the retention of HAC´s is also governed by the cylindrical contact surface between Ti-base and crown, the introduction of a sharp-edged groove may act as a stress concentrator, facilitating crack initiation, which can spread without restriction due to the circumferential groove design, and overall compromising uniform stress distribution along the adhesive interface. Instead of a homogeneous load transfer across the entire cylindrical contact surface, the MR may lead to mechanical disengagement. The change in layer thickness can cause the cement within the groove to lose its direct, compressed contact with the interface, thereby locally diminishing the adhesive´s effectiveness of the chemical bond. The positive effect on bond strength observed for rougher titanium surfaces can be attributed to an optimization of the micromechanical and chemical bonding mechanisms at the contact surface. Increased SB pressure leads to an enlarged surface, creating a retentive microtopography which can be penetrated more effectively by the low-viscosity bonding agent. At the same time, SB creates high kinetic energy, which may further disrupt the passive titanium layer and expose a highly reactive surface. In combination with the phosphate monomer-containing primers used, this may maximize chemical interaction.

The results of the conventionally pretreated groups without MR were consistent with those of previous studies using comparable materials [16, 17, 23]. In line with the present findings, authors reported mixed adhesive failure as the most frequent mode under standard pretreatment protocols [16, 17]. Direct comparison of related investigations is often limited by methodological heterogeneity. In the study by Elzami et al. [23], comparable pull-off tests were performed using Vita Enamic, following thermocycling of specimens (5000 cycles, 5/55°C) and SB of abutments with 50 μm particles. Although abutments were treated with the same particle size as applied in the control group of the present study, SB was conducted at 2 bar, corresponding to group H3, and different adhesive bonding systems were employed. Furthermore, the abutments used by Elzami et al. featured prefabricated retention grooves, resulting in a design that differs from the abutments utilized in the present investigation. In their study, Enamic HAC specimens demonstrated a mean pull-off force of 729.99 N and are therefore most comparable to group H3 of the present study, in which the Ti-base was airborne-particle abraded at 2 bars but using 250 μm particles. However, it remains unclear whether the comparable retention values can be attributed primarily to the applied pressure or whether differences in the adhesive system and abutment geometry contributed to the observed outcomes. Notably, the failure mode analysis reported by Elzami et al. was consistent with the findings of the present study [23]. The additional MR provided by a circumferential groove in the ceramic represented an experimental variable that has been insufficiently investigated. A recent comparable study placed two groove points on the inner ceramic surface prior to bonding to zirconia abutments and reported higher retention than in control group without groove [24]. In addition, there are studies evaluating the effect of auxiliary grooves on the retention of tooth supported crowns [25]. The circumferential groove investigated here differs from these designs in geometry and extent, which may result in differences in stress distribution and bond line thickness. The enhancement of bond strength associated with larger alumina particle sizes has also been reported in previous studies [5, 26].

The sample size used in the present investigation lies at the upper end of the range reported in comparable studies of comparable experimental design, where usually sample sizes between 8 and 15 are provided [3, 27–30]. The selected conditioning protocols were based on established recommendations and reflect common clinical practice. Etching of PICN crowns with HF and alumina SB for zirconia and titanium are standard surface conditioning methods. The crowns were specifically designed for tensile shear test. Deviating from a natural tooth, modifications were made to create standardized measurement conditions. Thermal aging of the specimens was performed using a thermocycling protocol that corresponds to procedures widely applied in comparable studies. The lower crown margin was widened and flattened, ensuring a uniform contact surface of the testing tool. This intended to reduce the risk of uneven force transmission during pull-off test. This type of specimen design has already been employed in previous studies [16, 17]. Although additive manufacturing of the crowns could have been used to incorporate MR features, the MR elements were instead attached post-fabrication. This approach was chosen because the predrilled holes in the material blocks and their corresponding adhesive bases are preassembled and matched, thereby minimizing fit inaccuracies. Tensile shear test is a well-established method in materials science for quantitative assessment of bond strength of adhesive interfaces. Maximum pull-off force (Fmax) recorded during testing serves as an indicator of mechanical stability of the bonded interface [31]. Testing was conducted under standardized conditions using a universal testing machine. To ensure defined, coaxial load application, a custom pull-off device was employed. Its articulated mounting permitted self-alignment of specimens along the tensile axis, minimizing parasitic bending and shear forces that could influence test results. Load application was performed at a constant crosshead speed of 1 mm/min, a parameter commonly used in comparable studies, allowing for precise determination of maximum pull-off force [31].

This study was limited to two restorative materials: zirconia and PICN HACs. Therefore, the findings cannot be transferred to ceramic systems with different microstructures, compositions, or mechanical properties. Other ceramics may respond differently to the surface pretreatment protocols evaluated here. Furthermore, the experimental design evaluated only one MR geometry and a single SB protocol. This raises the question of whether alternative MR designs could avoid the adverse effect of the circumferential groove. Possible options include point-shaped, rounded depressions or interrupted vertical grooves on the inner crown surface. Compared with a continuous groove, these geometries may reduce stress concentrations and limit crack propagation, while confining cement volume and polymerization shrinkage to localized areas. However, any macroscopic material reduction on the inner surface of crowns may involve material-specific risks. In the present study, modified SB parameters (2 bar, 250 μm) led to an increased overall bond strength. Nevertheless, the clinical implementation and the associated risk factors must be carefully considered. SB with a larger particle size and increased pressure may lead to higher substance loss at the titanium surface. As mechanical fit between the components is within a range of micrometres, uncontrolled material removal can compromise cement gap precision. This can raise the risk of decementation under functional loading. At higher blasting pressures, improper handling may also increase the risk of damaging the highly precise implant-side connection geometries or the anti-rotational features of the Ti-base. Another clinical risk is possible contamination. Due to high kinetic energy during blasting with 250 μm and 2 bar, there may be an increased embedding of Al₂O₃ particles in the relatively soft titanium surface. It remains unclear to what extent these residues can be removed by subsequent cleaning and to what extent these residues affect the chemical stability of the bond. Furthermore, the recommendations of the manufacturer and the associated warranty limitations must be considered. Most manufacturers recommend a maximum of 2 bar with 50 μm Al₂O₃ for surface pretreatment of Ti-bases. Increasing the grain size to 250 μm deviates fundamentally from these recommendations. In case of damage, this may result in the loss of manufacturer warranties and legal warranty claims. For these reasons, altering the protocol for clinical use cannot be recommended. Prior investigations are needed to thoroughly assess the effects of these modified parameters on the titanium surface and on fit accuracy.

Despite following established protocols, the absence of cyclic loading, as only thermocycling without mechanical cyclic loading was performed, represents a limitation of the present investigation, as it limits conclusions regarding long-term clinical performance. Preparation and grouping of specimens were based on comparable studies [24, 32, 33]. Since the investigation is an explorative in vitro study and materials were limited, the study was conducted without sample size calculation. Nevertheless, the sample size used in this investigation proved a sufficient volume for statistical comparisons among the evaluated surface pretreatment protocols and the calculated minimal detectable differences were within a range that can be considered clinically relevant. Nevertheless, an a priori sample size calculation could have improved confidence in statistical power.

Future research should expand the experimental framework to include additional retention concepts, alternative testing conditions, and a broader range of restorative materials. Recently developed additively manufactured ceramics should be incorporated into subsequent investigations to more comprehensively characterize their bonding behavior.

## Conclusions

In this in vitro study, CAD/CAM-fabricated PICN and zirconia HACs exhibited greater bond strength to Ti-bases when the titanium was SB at 2.0 bar with 250 μm alumina grit versus 1.0 bar and 50 μm grit, producing a demonstrably rougher surface. Incorporation of additional MR in form of a circumferential groove within the crown decreased bond strength relative to the standard procedure.

From a clinical perspective, adding additional MR as implemented here cannot be recommended, because they can weaken the overall mechanical performance of the system. Despite the deliberate increase of Ti-base surface roughness by increasing SB pressure or blasting with coarser alumina particles appears to be an effective method to enhance bond strength, unrestricted clinical application is not indicated as potential risks like surface damage would need to be investigated in more detail and, manufacturers’ instructions should be followed to maintain warranty coverage.

Overall, the results of this study indicate that optimizing SB parameters of Ti-bases offers further potential and appears more beneficial than adding the specific MR geometry tested to improve bond strength. Therefore, it may be useful to further investigate the previously described issues associated with intensified SB of the Ti-base.

## Data Availability

The datasets used and/or analysed during the current study are available from the corresponding author on reasonable request.

## Abbreviations

HAC: Hybrid abutment crown
CAD/CAM: Computer aided design/ computer aided manufacturing
Ti-bases: Titanium bases
PICN: Polymer-infiltrated ceramic networks
MDP: Methacryloyloxydecyldihydrogen-phosphate
RV: Roughness value
SB: Sandblasting
mm: Millimeters
MR: Macroretention
F_max_: Maximum pull-off force
HF: 5% hydrofluoric acid
mm/s: Millimeters per second
Ra: Arithmetic mean roughness
Rq: Quadratic mean roughness
Rz: Average roughness depth
s: Seconds
N: Newton
